# Synaptamide Modulates Astroglial Activity in Mild Traumatic Brain Injury

**DOI:** 10.3390/md20080538

**Published:** 2022-08-21

**Authors:** Arina Ponomarenko, Anna Tyrtyshnaia, Darya Ivashkevich, Ekaterina Ermolenko, Inessa Dyuizen, Igor Manzhulo

**Affiliations:** A.V. Zhirmunsky National Scientific Center of Marine Biology, Far Eastern Branch, Russian Academy of Sciences, Palchevskogo Str., 17, 690041 Vladivostok, Russia

**Keywords:** N-docosahexaenoylethanolamine, synaptamide, mild traumatic brain injury, astroglia, BDNF, biomarkers, SOD, nNOS

## Abstract

At present, the study of the neurotropic activity of polyunsaturated fatty acid ethanolamides (N-acylethanolamines) is becoming increasingly important. N-docosahexaenoylethanolamine (synaptamide, DHEA) is a highly active metabolite of docosahexaenoic acid (DHA) with neuroprotective, synaptogenic, neuritogenic, and anti-inflammatory properties in the nervous system. Synaptamide tested in the present study was obtained using a chemical modification of DHA isolated from squid *Berryteuthis magister* liver. The results of this study demonstrate the effects of synaptamide on the astroglial response to injury in the acute (1 day) and chronic (7 days) phases of mild traumatic brain injury (mTBI) development. HPLC-MS study revealed several times increase of synaptamide concentration in the cerebral cortex and serum of experimental animals after subcutaneous administration (10 mg/kg/day). Using immunohistochemistry, it was shown that synaptamide regulates the activation of GFAP- and S100β-positive astroglia, reduce nNOS-positive immunostaining, and stimulates the secretion of neurotrophin BDNF. Dynamics of superoxide dismutase production in synaptamide treatment confirm the antioxidant efficacy of the test compound. We found a decrease in TBI biomarkers such as GFAP, S100β, and IL-6 in the blood serum of synaptamide-treated experimental animals using Western blot analysis. The results indicate the high therapeutic potential of synaptamide in reducing the severity of the brain damage consequences.

## 1. Introduction

According to clinical studies, 80–90% of all TBI cases may be classified as a mild form (mTBI), but they represent a serious problem of modern health care due to the development of significant long-term consequences. Maladaptation of glial activation is the main cause of these pathological transformations. Glial cells play a critical role in brain remodeling during injury and secondary neuroinflammation development. Astrocytes are known to be sensitive to changes in the extracellular environment and serve as critical early responders to brain injury [1]. Under pathophysiological circumstances, astrocytes respond to brain injury through a variety of molecular and cellular mechanisms called reactive gliosis [1,2]. Structural and biochemical changes occurring in astrocytes during the CNS injuries have a wide variability depending on the severity of the injury. Reactive astrocytes activate the production of ATP and extracellular Ca^2+^ [3], recruit immune cells from the peripheral blood [4], regulate the permeability of the blood–brain barrier [5], participate in post-traumatic synapse remodeling [6], and also contribute to neuroprotection by activating the production of neurotrophins. In this regard, the impact on astroglia through pharmacological agents seems to be the most promising therapeutic treatment strategy of the mTBI consequences. Currently, research into various natural compounds as therapeutic agents for the treatment of CNS injuries is gaining popularity. These substances usually have less toxicity and interactions with other drugs [7,8]. To date, there is a massive amount of preclinical data showing the presence of a positive therapeutic effect of drugs containing ω-3 polyunsaturated fatty acids (PUFAs) and their derivatives in acute brain damage [9]. ω-3 PUFAs exhibit anti-inflammatory activity due to the synthesis of resolvins, protectins, and maresins (bioactive mediators with pro-resolvent properties) [10]. In addition, PUFAs stabilize the cell membrane, making it more resistant to damage and more amenable to nerve impulse conduction [11]. The most important pathological factor in TBI is the neuroinflammatory response, so the treatment of docosahexaenoic acid (DHA, 22:6n-3), which has anti-inflammatory properties, seems to be very promising. N-docosahexaenoylethanolamine (synaptamide, DHEA) is an endogenous endocannabinoid-like metabolite synthesized from DHA [12]. Synaptamide has a complex effect similar to that of DHA, but much more pronounced [13].

Our previous study demonstrated that synaptamide reverses the cognitive deficits caused by mTBI and also reduces post-traumatic neuroinflammation [14]. This study is a continuation of previous work and is aimed at a more detailed study of the synaptamide effects in rat TBI model.

## 2. Results

### 2.1. Synaptamide Increases Serum and Brain Levels of DHEA and AEA

**Figure 1:**
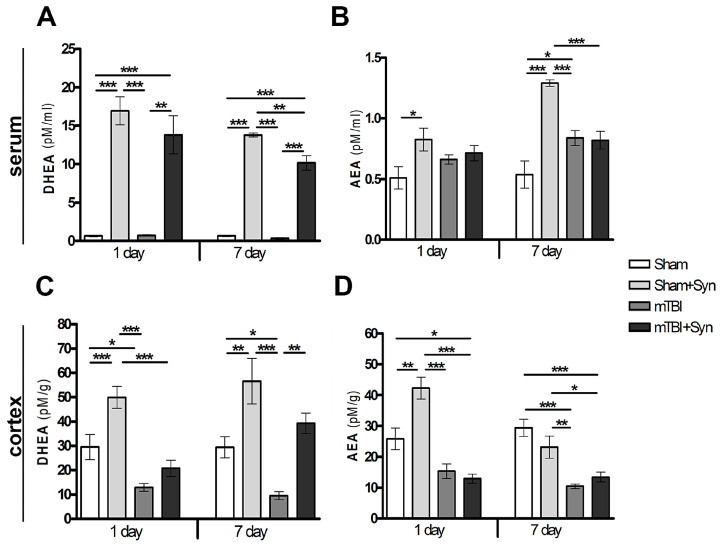
Quantitative content of NAE lipids in the cerebral cortex and blood serum of rats. DHEA, serum (**A**); AEA, serum (**B**); DHEA, cerebral cortex (**C**); AEA, cerebral cortex (**D**). Data are presented as mean ± SEM, * *p* < 0.05; ** *p* < 0.01; *** *p* < 0.001. n = 7/group (one-way ANOVA, Tukey’s post-test study).

Using the HPLC-MS method, it was found that synaptamide, even after a single subcutaneous injection demonstrates multiple increases in serum DHEA concentration. In this study, synaptamide was administered for 7 days after surgery. Day 1 and day 7 of the synaptamide administration were considered as control periods at which the measurement of the studied parameters took place. On the day 1 (in 24 h after the first injection), the level of DHEA rises by almost 25 times. A similar trend is observed on the 7th day after mTBI (Figure 1A). Interestingly, synaptamide therapy also affects serum concentration of N-arachidonylethanolamine (AEA), which doubles. However, under injury conditions, the synaptamide treatment have no influence on the AEA concentration (Figure 1B). mTBI changes the lipid composition of the brain lead to a decrease in DHEA (Figure 1C) and AEA concentration (Figure 1D). Surprisingly, the 7-day administration of synaptamide in the setting of trauma restore the cortex DHEA, but not AEA, levels to control values.

### 2.2. Synaptamide Reduces Serum Biomarkers after mTBI

**Figure 2:**
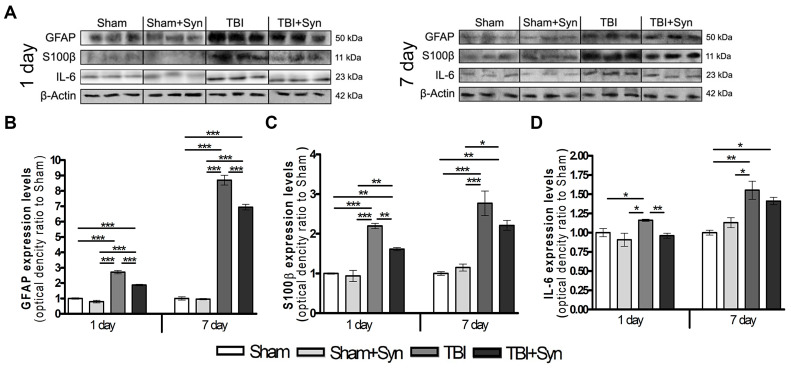
(**A**) Western blot analysis of GFAP, S100β, IL-6 proteins. Equal protein loading was determined using an β-Actin antibody. Effect of synaptamide on the expression of TBI biomarkers: (**B**) GFAP, (**C**) S100β, (**D**) IL-6. Data are presented as mean ± SEM, * *p* < 0.05; ** *p* < 0.01; *** *p* < 0.001. n = 7/group (one-way ANOVA, Tukey’s post-test study).

The use of blood biomarkers is a supplementary tool for assessing the severity of the brain damage and temporal profiles of the mTBI development. The main proteins expressed in the blood during TBI (GFAP, S100β and IL-6) were analyzed by Western blotting of the blood serum of experimental animals. Data are normalized to β-Actin. GFAP, a protein of intermediate filaments of the astrocytic cytoskeleton, has been successfully used to assess the severity of damage and predict outcomes in acute TBI [15]. Our data confirm the previously published results [16] on the increase in the GFAP concentration in the blood serum after mTBI. Moreover, there is a tendency to progression of GFAP expression and accumulation from 1 to 7 days. After 24 h, the concentration of serum GFAP exceeds the control values by 65%, and after 7 days of the experiment, this difference is about 88% (Figure 2A,D). Therapy with synaptamide, even with a single injection in animals with mTBI, induces a decrease in serum GFAP concentration relative to those in the “mTBI” group.

In many studies, S100β, a calcium-binding protein that is predominantly expressed in astroglia, applied to assess the extent of injury in the acute care clinical setting [17]. In the present work, Western blotting revealed a significant increase in the concentration of S100β protein in the blood serum of animals with mTBI compared to Sham-operated animals. We found an increase in the concentration of the S100β protein in the blood serum of animals with mTBI from day 1 to day 7, which indicates the progression of secondary brain damage a week after the injury induction (Figure 2B,D). Synaptamide therapy causes a significant decrease in the concentration of serum S100β on day 1 by 20%, and on day 7 the downward trend persists, but there is no significant difference between the “mTBI” and “mTBI+Syn” groups.

After TBI, both pro- and anti-inflammatory cytokines are upregulated and their combined activity determines the overall degree of inflammation [18]. Interleukin-6 is a pleiotropic cytokine that is a biomarker for various disease states, including TBI [19]. mTBI causes an increase in the IL-6 concentration in the blood serum both after 24 h (by 16%) and after 7 days of the experiment (by 55%) (Figure 2C,D). On the day 1, synaptamide therapy restored the studied parameter to control values, and after 7 days a decreasing tendency in the IL-6 concentration was seen; however, no significant difference was found between the “mTBI” and “mTBI+Syn” groups.

### 2.3. Synaptamide Regulates Reactive Astrogliosis in mTBI Conditions

**Figure 3:**
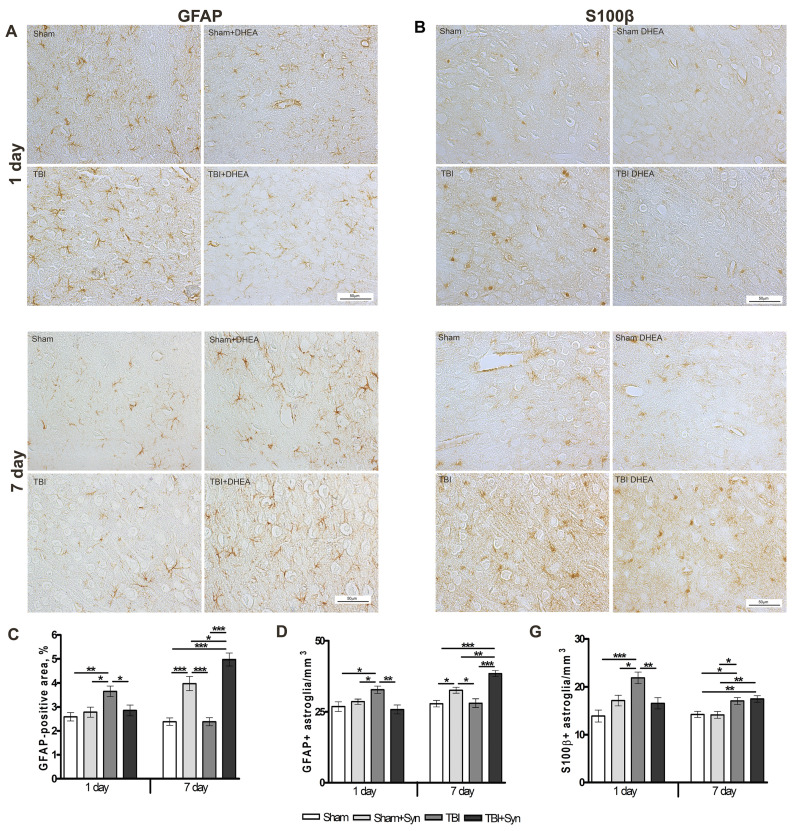
Effect of synaptamide on astroglial activation in mTBI. Immunolocalization of GFAP+ astroglia in the cerebral cortex of experimental animals. Scale bar: 50 μM. (**A**). Staining area of GFAP+ astroglia. (**C**). Quantitative distribution of GFAP+ astroglia (**D**). Immunolocalization of S100β+ astroglia in the cerebral cortex of experimental animals. Scale bar: 50 μM. (**B**). Staining area S100β+ astroglia (**E**). Data are mean ± SEM, n = 7/group, * *p* < 0.05, ** *p* < 0.01, and *** *p* < 0.001 (one-way ANOVA, Tukey’s post-test study).

Astroglia is involved in the pathogenesis of excitotoxicity and cytoprotection in CNS lesions. After mild or diffuse TBI, as well as in the perifocal zones around focal CNS lesions, large areas of functioning tissue contain astrocytes in a state of reactive gliosis [20]. Evaluation of GFAP+ immunostaining showed an increase in astroglial activity in the ipsilateral cerebral cortex of animals with mTBI on the first day of the experiment (Figure 3A). After 24 h, both the area of immunostaining and the number of GFAP+ cells per mm^3^ increase, while synaptamide therapy reduces astrogliosis to the level of Sham-operated animals (Figure 3C,D). At the same time, on the day 7 in the groups of animals treated with synaptamide, astroglial activity reached the level of the “Sham” group (Figure 3A,C,D).

As an intracellular regulator, another astrocytic marker S100β stimulates cell proliferation and migration, inhibits apoptosis and differentiation, as well as astrocyte activation, which may be important for brain recovery after CNS damage [21]. Figure 3B,E demonstrates that S100β production is increased after mTBI at both periods of the experiment. Synaptamide inhibiting the expression of S100β in astrocytes for a period of 24 h does not have a similar effect after 7 days.

### 2.4. Synaptamide Reduce nNOS Production and Contributes to the Antioxidant Protection of Neurons

**Figure 4:**
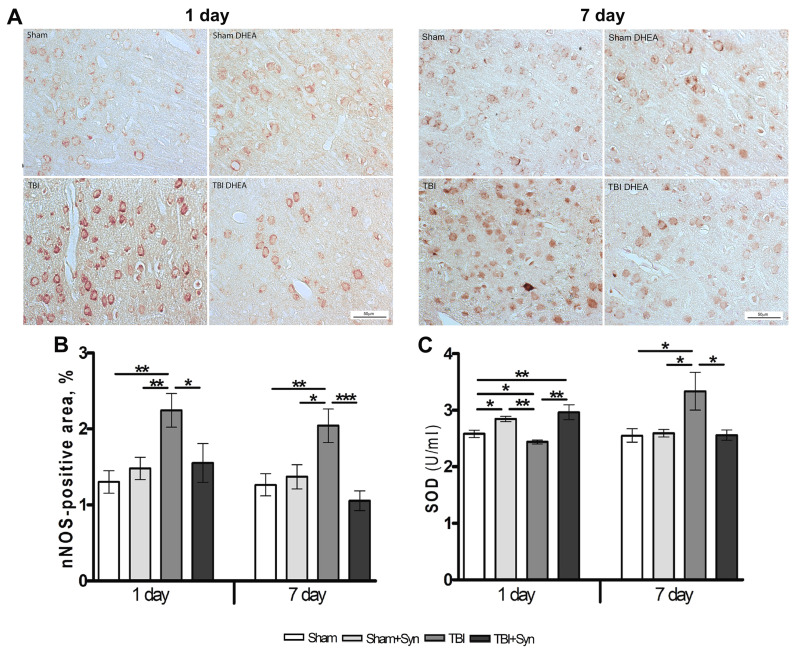
Expression of nNOS in the cerebral cortex of rats. Scale bar: 50 μM. (**A**). Staining area of nNOS+ neurons in the cerebral cortex (**B**). SOD production in the cerebral cortex of rats with mTBI and synaptamide therapy (**C**). Data are mean ± SEM, n = 7/group, * *p* < 0.05; ** *p* < 0.01; *** *p* < 0.001 (one-way ANOVA, Tukey’s post-test study).

Neuronal nitric oxide synthase (nNOS), a local neuronal messenger and neuromodulator, is widely expressed in nervous tissue [22]. In CNS trauma, nNOS is the main effector of NMDA receptor-mediated excitotoxicity pathways [23], and is also an inducer of DNA damage due to enhanced production of nitric oxide (NO) and its highly reactive metabolite peroxynitrite [24]. The data of immunohistochemical studies demonstrate the effect of synaptamide on the nNOS production in the rat brain (Figure 4A). The area of nNOS+ neurons immunostaining doubles in both periods of the experiment. Synaptamide reduces nNOS synthesis almost to control values (Figure 4B).

After TBI, a complex of oxidative stress markers is produced in the brain (carbonylated proteins, lipid peroxides, reactive oxygen species and reactive nitrogen species), while the synthesis of antioxidant defense enzymes (GSH, GPx, GR, GST, G-6PD, SOD, CAT) is reduced. One of the most important endogenous antioxidants is the enzyme superoxide dismutase (SOD), which rapidly catalyzes the dismutation of O^2−^ into H_2_O_2_ and oxygen. It has been established that mTBI causes a significant decrease in the SOD enzyme on day 1, and synaptamide, after the first injection, increases its production even above the level of Sham-operated animals. However, after 7 days, the administration of synaptamide does not affect the synthesis of the enzyme, while mTBI causes a significant increase in SOD (Figure 4C).

### 2.5. Synaptamide Regulates Trophic Support of Neurons in mTBI through the Production of Brain-Derived Neurotrophic Factor (BDNF)

**Figure 5:**
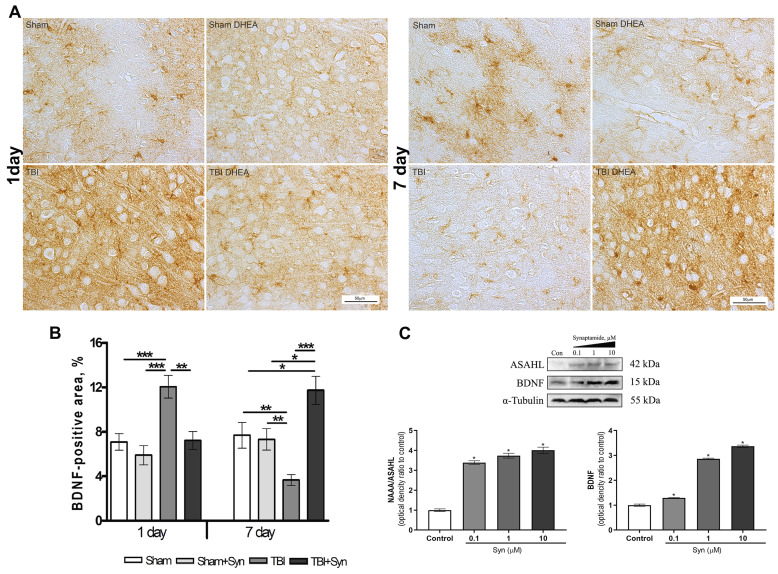
Immunolocalization of BDNF+ cells in the cerebral cortex of rats. Scale bar: 50 μM. (**A**) Staining area of the BDNF+ cells in the cerebral cortex. (**B**) Data are mean ± SEM, n = 7/group, * *p* < 0.05, ** *p* < 0.01, and *** *p* < 0.001 (one-way ANOVA, Tukey’s post-test study). (**C**) Western blot analysis of BDNF and NAAA/ASAHL levels in primary culture of astroglia. * *p* < 0.05, Significant differences between the “control” and “Syn” groups. Data are presented as mean ± SEM, n = 9 (number of samples analyzed), (pairwise comparison, Student’s *t*-test). Data normalized to α-tubulin.

Synaptamide is hydrolyzed to DHA and ethanolamine by fatty acid amide hydrolase (FAAH) and N-acylethanolamine-hydrolyzing acid amidase (NAAA/ASAHL) [25]. At the same time, NAAA/ASAHL is a lysosomal enzyme, while FAAH is an enzyme localized in the endoplasmic reticulum [26]. It has been established that inhibition of NAAA/ASAHL affects the development of neuroinflammation to a greater extent than inhibition of FAAH [27]. NAAA/ASAHL is highly expressed in macrophages, especially alveolar macrophages, and in peripheral tissues. Our data show that astrocytes also use NAAA/ASAHL to neutralize excess synaptamide. The results of Western blotting indicate a dose-dependent increase in the concentration of the enzyme when synaptamide is added to the culture medium (Figure 5C).

BDNF is one of the most widely distributed and well-studied neurotrophins in the mammalian brain. The effect of synaptamide on BDNF protein expression was assessed by Western blotting of a primary culture of astrocyte cells. The data shown in Figure 5C indicate an increase in the synthesis of neurotrophin, depending on the increase in the concentration of synaptamide. The immunolocalization of the BDNF protein in the rat cerebral cortex is shown in Figure 5A. The BDNF+ cell pool is heterogeneous and includes both neurons and glial cells. Immunohistochemical staining demonstrates no effect of synaptamide therapy on BDNF synthesis in the rat brain on the 1st day of the study; however, there is a significant increase in immunoreactivity induced by mTBI. On the 7th day after injury, the synthesis of neurotrophin in the brain of animals from the mTBI group, on the contrary, decreases compared to the Sham-operated animals. At the same time, under the injury conditions, the synaptamide treatment sharply increases the zone of BDNF-immunopositive staining (Figure 5B).

## 3. Discussion

Previously, we have already shown that synaptamide therapy in mTBI causes an improvement in cognitive functions, such as the level of anxiety and long-term memory, as well as a decrease in the development of post-traumatic neuroinflammation mediated by specific pro-inflammatory activation of microglia and the synthesis of pro-inflammatory cytokines and oxidative damaging mediators, such as NO, ROS, and nitrites [14]. In the present study, we explored the repair mechanisms implemented by modulating astrogliosis, the production of neurotrophins, nitric oxide, and enhancing the antioxidant defense of the brain during the treatment of mTBI with synaptamide. Synaptamide when administered subcutaneously (dose 10 mg/kg) has good bioavailability. Lipid chromatography data suggest that there are multiple increases in DHEA in rat serum after a single injection. There is also a slight increase in AEA on the 1st and 7th days of experiment, but only in the group of Sham-operated animals. Probably, the concomitant increase in AEA occurs due to the redistribution of the NAAA/ASAHL enzyme toward DHEA substrate. The activity of this enzyme increases in the primary rat astrocytes when synaptamide is added to the culture medium. Synaptamide therapy also increases the concentration of DHEA and AEA in the cerebral cortex. However, it is interesting that the injury itself causes a sharp decrease in NAE in the brains of animals after mTBI. There is evidence to support a decrease in endocannabinoid levels in the ipsilateral cortex at day 155 after severe TBI [28]; however, this effect in mild TBI was described for the first time.

Currently, many clinical studies confirm the importance of serum biomarkers in characterizing the severity and temporal profiles of TBI development. They allow tracking progression, predict clinical outcomes, and provide information on current pathophysiological changes [29]. In the present study, plasma protein S100β, GFAP, and IL-6 are detected in serum and show clear time profiles during the week after mTBI, which is consistent with earlier biomarker studies in TBI [30]. Treatment with synaptamide, apparently reducing the severity of damage, inhibits the production of mTBI biomarkers.

Micro- and astroglia play a crucial role in the adaptation of the brain to damage and secondary neuroinflammation development. Neuron-glial interaction is aimed at homeostasis maintaining, viable cells protecting, and cell debris utilizing. Reactive astrogliosis influences various processes that occur after injury, including (1) regulation of inflammation, (2) isolation of injury and protection of adjacent neural tissue, (3) regulation of blood-brain barrier permeability, and (4) modulation of synaptic plasticity and neural circuit reorganization [1]. Astrocytes contain dense networks of intermediate filaments such as GFAP. Mechanical damage associated with TBI disrupts the integrity of these networks and serves as a trigger for astrocyte deformation, determining the appropriate course of reactive astrogliosis and triggering the upregulation of GFAP and other intermediate filaments [31]. Our immunohistochemical data indicate an increase in GFAP expression on the 1st day after injury. However, by the 7th day in the “mTBI” group, both the area and the amount of GFAP+ astroglia decreased to the control level, which may indicate the depletion of adaptive capabilities. During the initial stages of damage, nuclear factor-kappa B (NF-κB) is activated in reactive astrocytes, leading to the production of pro-inflammatory factors such as tumor necrosis factor α (TNFα), α-chemokines, cyclooxygenase-2, and matrix metalloproteinase 9 [32]. Astrocyte-driven neuroinflammation is maintained by other cell populations, such as microglia/macrophages, as well as lymphocytes recruited from the peripheral blood. Our previous studies demonstrate an increase in the inflammatory activity of microglia and the synthesis of pro-inflammatory cytokines only on the 7th day after surgery [14], while on the first day microgliosis is not so pronounced [33]. In the delayed period of TBI, astrocytes perform a rather neuroprotective function by secreting neurotrophins and participating in synapse remodeling through the production of extracellular matrix molecules [34]. The depression of GFAP+ astroglia observed in mTBI is leveled out by synaptamide therapy. After 7 days, the proliferation of GFAP+ cells is activated both in the “Sham+Syn” and “mTBI+Syn” groups. Whereas the inflammatory component of secondary brain damage is reduced during synaptamide therapy [14].

Another astrocytic marker S100β is a functional protein that exhibits its activity depending on the concentration of extracellular calcium [35]. A significant increase in extracellular calcium caused by mitochondrial dysfunction, disruption of ion channels, or brain cell death in TBI triggers astrocyte transformation and activation, as well as the synthesis of S100β. In the present study, the amount of S100β+ astroglia in trauma doubled on day 1 of the experiment, and decreased by day 7, but remained significantly higher than the control values. Synaptamide suppresses S100β synthesis 24 h after injury; however, after 7 days, no significant difference was found between the “mTBI” and “mTBI+Syn” groups. Probably, in this case, synaptamide affects astroglial cells by inhibition of neuroinflammation, a decrease in glutamate production and excitotoxicity, reducing concentration of extracellular calcium, and preventing cell damage and death [3,4,36].

NF-κB, a transcription factor that induces the expression of several pro- and anti-apoptotic genes, is one of the major triggers for inflammation [37]. TNFα and NMDA receptor activation induces NF-κB-mediated increase in BDNF synthesis [38]. Increased BDNF concentrations trigger neuroprotective mechanisms in cortical and hippocampal neurons [39,40]. In the presented study, it was found that on the first day after injury, the number of BDNF+ cells increase sharply compared to the “Sham” group, while after 7 days, the adaptive capacity is probably depleted and BDNF synthesis decreases. It is known that chronic activation of NF-κB through the expression of a constitutively active form of the kinase NF-κB (IKK2) inhibitor beta-subunit, leading to neuroinflammation and decrease in the BDNF level, which correlates with cognitive impairment and neurodegeneration. Notably, when chronic NF-kB activation is turned off, BDNF expression and neurogenesis are restored [41]. Synaptamide increases the level of cAMP in cells and inhibits the translocation of the RelA subunit (p65—the main transcription factor of the NF-κB pathway) into the nucleus [42]. The addition of synaptamide at concentrations of 0.1–10 μM leads to a dose-dependent increase in BDNF synthesis in primary astrocytes. Synaptamide also enhances the synthesis of BDNF in vivo in the cerebral cortex of experimental animals. On the 7th day of the experiment, the staining area of BDNF+ cells in the “mTBI+Syn” group significantly exceeded that of the “mTBI” group. Our results are supported by the literature data that describe improvements in BDNF-mediated physiological functions such as learning and memory in TBI rats supplemented with DHA [9].

Over-activation of astrocyte N-methyl-D-aspartic acid receptors (NMDARs) and associated activation of nNOS play a critical role in neuronal death in TBI [43]. The mechanism of cell damage is associated with the aberrant activity of nNOS, leading to the formation of a large amount of peroxynitrite. Production of neuronal peroxynitrite in TBI causes calpain activation and consequently damage to neurons and their axons [44]. Our data indicate that nNOS production increases on the 1st day after injury and remains above control values for a week. Synaptamide reduces the production of nNOS at both periods of the experiment, thereby protecting neurons from the toxic effects of NO and peroxynitrite. Another mechanism of neuronal oxidative damage is based on excessive production of ROS and depletion of the endogenous antioxidant system, which causes peroxidation of cellular and vascular structures, protein oxidation, and inhibition of the mitochondrial electron transport chain [45]. The enzyme SOD, which converts O^2−^ to H_2_O_2_ and oxygen, has been extensively studied and demonstrates therapeutic potential in a variety of disease models exhibiting excitotoxicity, including an in vitro of NMDA-induced neuronal culture [46] and in vivo model of middle cerebral artery occlusion in rats [47]. In addition, the SOD enzyme, through the regulation of redox balance, plays a critical role in various biological processes, including inflammation, autophagy, and cell survival [48]. The presented data indicate an endogenous SOD concentration increase in 24 h after synaptamide administration in both Sham-operated and injured animals. This is consistent with the previous data obtained in the primary culture of astrocytes with the addition of synaptamide at a concentration of 10 μM [49]. However, in “mTBI” group, the growth in SOD activity is observed one week after the surgery, when neuroinflammation was most pronounced [14]. At the same time, the effect of treatment on the 7th day was not pronounced. Probably, synaptamide, reducing neuroinflammation, creates conditions for oxidative balance, in which there is no necessity for hyperproduction of SOD, which remains at the level of Sham-operated animals in the “mTBI+Syn” group. The data obtained are consistent with our previous results, where the ability of synaptamide to reduce the level of microgliosis and pro-inflammatory cytokines in mTBI, as well as ROS, NO, and nitrites production in LPS-induced inflammation was reveled in microglial cell culture [14].

The results of this study indicate the wide range of synaptamide glial-based and neuroprotective effects, emphasizing its high therapeutic potential.

## 4. Materials and Methods

### 4.1. N-Docosahexaenoylethanolamine (DHEA, Synaptamide)

Squid (*Berryteuthis magister*) was fished in the western part of the Bering Sea at a depth of 400–600 m. Then a concentrate of polyunsaturated fatty acids was obtained from the liver of squid according to the method of Latyshev et al. (2014) [50]. Further, ethyl esters of PUFA were isolated to obtain ethanolamines of fatty acids (US Patent 3,257,436). HPLC of ethanolamines of polyunsaturated fatty acids was performed on a Shimadzu LC-8A chromatograph (Shimadzu, Kyoto, Japan) with UV/VIS SPD-20A (205 nm). Separation was performed on a Supelco Discovery HS C-18 preparative reverse phase column (Bellefonte, PA); particle size 10 μm, inner diameter 250 mm × 50 mm; and isocratic elution was used with an ethanol/water system (70:30, *v*/*v*), elution rate 50 mL/min. Fractions containing synaptamide were collected, evaporated in vacuo, and analyzed by GC and GC-MS. DHEA was a light-yellow liquid oil with a mild odor at room temperature and had a purity of 99.4%.

### 4.2. Animals

Male Wistar rats (260 ± 20 g, age 3 months) were used in experiments. Females were excluded from the study to avoid the influence of ovarian cycles. The rats were kept for 2–4 persons in a cage with unlimited access to food and water. The animals were maintained at a constant temperature (23 ± 2 °C) and humidity (55 ± 15%) with a 12-h light/dark cycle (light at 7:00). All animals were divided into four groups: the “Sham” group (n = 14); group “Sham+Syn” (n = 14)—animals injected with synaptamide; group “mTBI” (n = 14)—animals with mild traumatic brain injury; group “mTBI+Syn” (n = 14)—animals that received mTBI, treated by synaptamide. The animals were withdrawn from the experiment, with 7 individuals from each group on the 1st and 7th days after mTBI. The experiment was repeated twice to collect material for immunohistochemical and biochemical studies. All procedures were approved by the Animal Ethics Committee at the A.V. Zhirmunsky National Scientific Center of Marine Biology, Far Eastern Branch, Russian Academy of Sciences (No 06/2020, approved on 15.06.2020), and carried out in accordance to the international regulations of the European Directive 2010/63/EU.

### 4.3. mTBI Procedure

General anesthesia was performed with isoflurane inhalation anesthetic (Laboratories Karizoo, Barcelona, Spain) (4.5%) in 100% oxygen (VetFlo, Kent Scientific Corporation, Torrington, CT, USA) [51] for approximately 1–2 min, maintained through the nasal cone. After the extinction of the main reflexes, a midline skin incision over the skull was performed, and the skull was exposed to locate the area of impact. Mild TBI was formed on a weight-drop device (Northeast Biomedical Inc., Tyngsborough, MA, USA, cat. 2700) by dropping a metal rod weighing 337 g with a blunt end from a height of 8 cm onto the scalped skull. The impact site was in the right hemisphere, 2 mm lateral to the midline and 4 mm forward from the lambdoid suture. After surgery, the skin was sutured and treated with an antibacterial spray to prevent infection. In the “Sham” groups, the animals underwent the same surgery as described above, with the exception of mTBI. Immediately after surgery, the animals were injected subcutaneously with synaptamide solution or saline. An emulsion of synaptamide was prepared by dissolving in 0.9% saline. Each rat was then placed to recover on a warm pad until thermoregulation and an alert state was reestablished, and then returned to their home cages, with free access to food and water. During the next week, animals in the “mTBI+Syn” and “Sham+Syn” groups were injected daily subcutaneously with emulsion of synaptamide at a dose of 10 mg/kg, while animals in the “Sham” and “mTBI” groups were injected with 0.9% saline in the same mode.

### 4.4. Immunohistochemistry and Microscopy

Brain tissue was harvested 1 or 7 days after mTBI. Rats (n = 7 animals/group) were anesthetized with 4.5% isoflurane in 100% oxygen and transcardially perfused with 20 mL of ice-cold saline followed by 20 mL of cold fixative (4% paraformaldehyde in 0.1 M phosphate buffer (PBS), pH 7.2). The brain was then immediately removed and placed in fresh buffered 4% paraformaldehyde for 24 h at 4 °C. After washing in PBS, the brain was processed and embedded in paraffin according to standard methods. Paraffin sections (7 μm) were obtained from the brain segment located immediately below the target area (Bregma −4.56 mm). Then, the tissue samples were embedded in paraffin blocks and coronal sections with a thickness of 7 μm were made using a Leica rotary microtome (RM 2245; Leica Biosystems, Buffalo Grove, IL, USA).

After dewaxing and incubation in H_2_O_2_ (3%), the sections were placed in blocking buffer containing 2% bovine serum albumin (sc-2323; Santa Cruz Biotechnology, Santa Cruz, CA, USA) and 0.25% Triton X-100 (Sigma, St. Louis, MO, USA) for 1 h RT. Incubation with primary antibodies was carried out overnight at 4 °C. Negative control was also performed (without primary antibodies). The following primary antibodies were used for immunohistochemistry: GFAP (mouse monoclonal, 1:2000, AMAb91033, Sigma Aldrich, St. Louis, MO, USA), S100β (rabbit polyclonal, 1:1000, ab41548, Abcam, Cambridge, UK), BDNF (rabbit monoclonal, 1:1000, Abcam, ab108319, Cambridge, UK), nNOS (rabbit monoclonal, 1:1000, Millipore, 07-571-I, Temecula, CA, USA). Appropriate secondary antibodies conjugated with horseradish peroxidase (PI-1000, anti-rabbit; PI-2000, anti-mouse) were used according to the manufacturer’s instructions (Vector Laboratories, Burlingame, CA, USA). After washing, the sections were treated with a chromogen (TL-060-QHD, Thermo Scientific, Burlingame, CA, USA) for 5–10 min to induce an immunoperoxidase reaction. The sections were then washed with distilled water, dehydrated and mounted onto slides using mounting medium (CS705, Dako, Denver, CO, USA).

Images were acquired with a size of 530 × 710 μm using a dry objective × 20 NA 0.45 (Plan-Apochromat) on a microscope (Axio Image Z2, Carl Zeiss, Oberkochen, Germany) equipped with a CCD camera (AxioCam HRc) (Carl Zeiss, Oberkochen, Germany). For the analysis, at least 70 images of the ipsilateral cerebral cortex were used for each group of animals. All measurements were performed by an operator who does not know the identity of the sections.

### 4.5. Assessment of SOD Enzyme Activity

An additional cohort of animals was taken for the assessment of SOD enzyme activity. The animals were anesthetized, after which each brain was quickly extracted, frozen in liquid nitrogen, and stored at −70 °C until use. SOD activity was determined in homogenates of the cerebral cortex of experimental animals using a commercial kit (Sigma Aldrich, 19160, St. Louis, MO, USA) and calculated as U/1 mg of protein. A calibration curve was built using superoxide dismutase (Sigma Aldrich, S9697, St. Louis, MO, USA).

### 4.6. Cells for Bioassay

**Figure 6:**
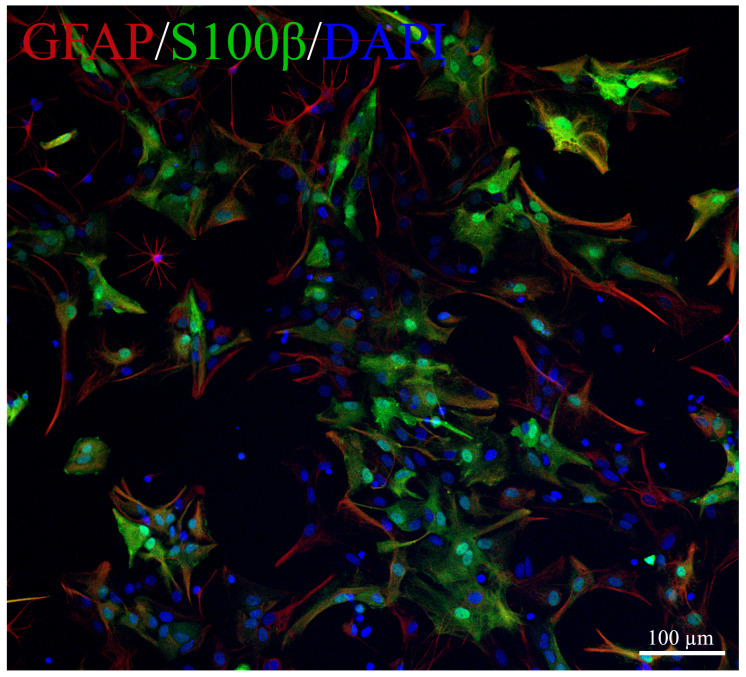
Representative images of primary culture of astroglia. GFAP-red, S100β-green, DAPI-blue.

To determine the activity of the NAAA/ASAHL and BDNF, we used a primary culture of astrocytes isolated from 1–2 days old rat pups, obtained in accordance with the protocol by Schildge et al. (2013) [52]. Briefly, on day 7, to obtain a pure astrocyte culture, the mixed cell culture was shaken at 180 rpm for 30 min on an orbital shaker to remove microglia, and then shaken at 240 rpm for 6 h to remove the oligodendrocyte precursor cells. Then astrocytes were cultured for 14 days in standard culture medium DMEM/F12 (Thermo Fisher Scientific, Waltham, MA, USA) with 10% fetal bovine serum (Thermo Fisher Scientific, Waltham, MA, USA), essential amino acids (Gibco, Waltham, MA, USA), and penicillin-streptomycin (Thermo Fisher Scientific, Waltham, MA, USA) in an incubator (MCO-18AIC, Sanyo, Osaka, Japan) at 5% CO_2_ 37 °C. Trypsin-EDTA (0.05%) (Thermo Fisher Scientific, Waltham, MA, USA) was applied for 5 min for cell passaging. To confirm the purity of the astroglia cell culture, immunocytochemical analysis was performed by staining with GFAP (mouse monoclonal antibody, 1:2000, Sigma Aldrich AMAB91033, Saint Louis, MO, USA) and S100β (rabbit polyclonal, 1:1000, ab41548, Abcam, Cambridge, UK) (Figure 6). For Western blotting, astroglial cells were plated in 6-well plates (1 × 10^5^ cells/well) and incubated with 5% CO_2_ at 37 °C overnight. Then the standard medium was replaced with a medium containing 10 μM synaptamide. Cells cultured in standard culture medium were used as controls. Incubation with synaptamide lasted 2 days, then astrocytes were carefully passed using a trypsin-EDTA solution, centrifuged, the supernatant was removed, and the pellet was frozen in liquid nitrogen until further use.

### 4.7. Biomarker Measurement

Serum levels of GFAP, S100β, and IL-6 were measured by Western blotting to assess the effect of synaptamide on the severity of mTBI. The rats (n = 7 animals/group) were anesthetized, then the chest was opened, and blood was immediately withdrawn from the left ventricular cavity using a syringe. Then, each blood sample was transferred to an individual serum tube (Greiner Bio One, Kremsmünster, Austria), coated with a clotting activator, and centrifuged. The collected supernatant was frozen in liquid nitrogen until further use.

### 4.8. Western Blotting

Western blotting was used to detect NAAA/ASAHL enzyme levels in primary astrocytes treated with DHEA and serum levels of GFAP, S100β, and IL-6 in experimental animals after mTBI. Samples were subjected to ultrasonic homogenization in dilution buffer (PBS) supplemented with 0.150 mM serine protease inhibitor (PMSF). After that, the concentration of the protein was measured with subsequent leveling to values of 2 mg/mL. The samples were then diluted 1:1 with a stock loading buffer (1× Sample buffer—Biorad, Hercules, CA, USA) containing 5% 2-mercaptnoethanol, and then placed in a water bath at 94.5 °C for 5 min. Electrophoresis was performed using the Biorad system, using ready-made Protean mini gel Any kDa gel cartridges (Biorad, Hercules, CA, USA) and Spectra Multicolor Broad Range Protein Ladder (Thermo Fisher Scientific, Waltham, MA, USA), the load per well was 60 μg of protein, the current strength per gel was 15 mA. After electrophoresis, proteins were transferred onto a PVDF membrane using a Turbo transblot transfer system (Biorad, Hercules, CA, USA). All transfer materials were used from the Transblot Turbo RTA Transfer kit (Biorad, Hercules, CA, USA). Upon completion of the transfer, the membranes were placed in blocking buffer (1× phosphate buffer containing 2% BSA, 0.1% Tween20, 0.05% TritonX100) overnight. The next day, the blocking buffer was washed with PBS + 0.1% Tween20, after which it was incubated for 1 h with primary antibodies. Primary antibodies to the NAAA/ASAHL (1:1500, Santa Cruz Biotechnology, sc100470, Santa Cruz, CA, USA) and BDNF (1:1000, Abcam, ab108319, Cambridge, UK), were used for the astrocyte cell culture. Primary antibodies to GFAP (1:2000, Sigma Aldrich, AMAb91033, St. Louis, MO, USA), S100β (1:1000, Abcam, ab41548, Cambridge, UK), and IL-6 (1:1500, Abcam ab208113, Cambridge, UK) were used for blood serum. Moreover, antibodies to α-tubulin (1:1000, Thermo Fisher Scientific, A11126, Waltham, MA, USA) and β-actin (1:5000, Thermo Fisher Scientific, MA1-140, Waltham, MA, USA) were used as load control. After incubation with primary antibodies, the membranes were washed again with PBS-T, then incubated for an hour with secondary antibodies Anti-Rabbit (Abcam, Cambridge, UK) and Anti-Mouse (Abcam, Cambridge, UK). Western Blot ECL Substrate (Biorad, Hercules, CA, USA) was used to carry out the chemiluminescence reaction (1 mL of substrate per membrane, 5 min). Visualization was performed using the ChemiDoc gel documentation system (Biorad, Hercules, CA, USA). The resulting images were analyzed using the ImageLab software package.

### 4.9. Quantitation of NAE in Brain Lipids

The cerebral cortex of rats (n = 7 animals/group) were taken for the analysis of N-acylethanolamines (NAE). The animals were anesthetized, after which each brain was quickly extracted, frozen in liquid nitrogen, and stored at −70 °C until use. Internal standard 0.1 nM (22:0-NAE) was added to each rat brain sample, followed by lipid extraction according to the method of Bligh and Dyer (1959) [53]. The final lipid residue was reconstituted in 1 mL of ice-cold CHCl_3_, purged with argon, and stored at −70 °C until high performance liquid chromatography-mass spectrometry (HPLC-MS) was performed. NAE analysis by HPLC-MS was achieved by Ascentis C18 analytical column (2.1 292 mm × 100 mm × 3-micron, Supelco, Bellefonte, PA, USA) and LC-MS 8060 mass spectrometer (Shimadzu, Kyoto, Japan). Mass spectra were obtained during ionization in an electric field in the positive ion detection mode. Mixtures were analyzed by multiple reaction monitoring (MRM). The ion source parameters were set at: interface temperature 380 °C; desolvation line temperature 250 °C; nebulizing gas (N2) flow—3 L/min; drying gas (N_2_) flow—3 L/min; and heating gas (dry air) flow—17 L/min. Collision energy was optimized for each compound individually. The molecular ion and fragment for each compound measured were as follows: 348→62 for AEA, 372→62 for DHEA, and 384→62 for the internal standard (22:0-NAE). Quantification of each NAE in the tissue samples was achieved using LabSolution (Shimadzu, Kyoto, Japan) followed by comparison of the peak area with the internal standard peak area.

### 4.10. Statistical Analysis

Data were subjected to statistical analysis using one-way ANOVA tests followed by a post hoc Tukey’s multiple comparison test. Data were shown as mean ± SEM, and *p* < 0.05 was regarded as statistically significant. All statistical tests were performed using the GraphPadPrism 4.00 software (GraphPad Software, San Diego, CA, USA).

## Data Availability

The datasets used and analyzed during the current study are available from the corresponding author on reasonable request.
